# Sports and Special Populations: Training Physiology in Health and Sports Performance

**DOI:** 10.3390/healthcare11152198

**Published:** 2023-08-04

**Authors:** Felipe J. Aidar

**Affiliations:** 1 Graduate Program of Physiological Science, Federal University of Sergipe (UFS), São Cristovão 49100-000, Brazil; fjaidar@gmail.com; 2 Group of Studies and Research of Performance, Sport, Health and Paralympic Sports (GEPEPS), Federal University of Sergipe (UFS), São Cristovão 49100-000, Brazil; 3 Graduate Program of Physical Education, Federal University of Sergipe (UFS), São Cristovão 49100-000, Brazil

Health is increasingly being studied, notably concerning preventive measures for health issues [1,2,3]. Problems related to modern lifestyles, particularly the effects of hypokinesia, have been a focal point in recent research [4,5]. Therefore, engaging in regular exercise along with ensuring a healthy diet, among other factors, are believed to play a decisive role in promoting health [1,4]. In this sense, engagement in sports would positively impact health indicators [3,5]. However, several factors influence athletes’ success, rehabilitation, and physical preparation [6,7].

Furthermore, parasport and special populations have been extensively researched, and the performance and health of these populations have been affected by the relationship between stress, maturation, training load, and recovery [8,9]. Additionally, environmental approaches aiming to enhance efficiency and physiological adaptation in relation to training and intervention methods are being explored [10,11]. However, in various situations and conditions, the stimulus–performance–adaptation relationship may vary and can interfere with recovery, performance, and health [12,13].

Thus, topics such as training load monitoring; stress and physiological responses exhibited during exercise or sports; post-exercise recovery processes; alterations due to stress and/or training load; and the physiology of training in health and athletic performance could significantly impact health. In this context, 19 published studies related to the subject have been identified.

We begin with stress assessment, where the influence of slow breathing on acute stress among handball coaches was evaluated. It was indicated that implementing respiratory control could improve stress conditions during official games, with potential impacts on health [14].

Another study investigated the impact of sleep deficiency on oxidative stress, PCR-us, and cortisol levels associated with different intensities of aerobic exercise. Exercises of varying intensities were compared, and the results suggested that lower exercise intensity was more effective in mitigating the negative effects of sleep deficiency [15].

Furthermore, one study compared resistance training based on speed and percentages. Training based on percentages was found to be more effective in maintaining resistance to high-power speed, while speed-based training had a greater impact on explosive power adaptations [16].

Another focus of research was on individuals with disabilities, including people with Down’s syndrome, and their relationship with training and swimming. The body composition and physical restriction profiles of competitive swimmers and moderately active (detrained) individuals with Down’s syndrome were compared. The results indicated that competitive swimming had a positive effect on reducing the tendency toward obesity and improving the strength, efficiency, and balance of this population [17].

Aquatic activities were also evaluated with respect to the health, psychological, and social indicators in people with disabilities. Significant improvements were observed in the mentioned conditions, as well as in social relationships and the inclusion of individuals with disabilities, with increased support for their parents [18].

Furthermore, concerning individuals with disabilities, a scale was developed to predict the pre-service intentions of physical activity instructors for people with disabilities. The scale addressed the constructs, followed by the behavioral beliefs, that were influenced by teaching experience regarding physical activity for people with disabilities. The influence of parents and close associates of individuals with disabilities, as well as the beliefs of those involved in teaching physical activity to this group, were also observed [19].

The relative importance and priority of factors to be considered in the administration of physical activities for people with disabilities were also evaluated. The type of facility and the provider of information were considered highly important, and it was observed that programs within sports facilities had a higher priority. The utilization of these locations by individuals with disabilities seems to be a crucial point outlined by this approach [20].

In the realm of parasports, studies were focused on various sports. Notably, the Paralympic Games (PG) are considered one of the world’s largest events, with increasing media coverage and participation. One study aimed to investigate the variation in the number of gold, silver, bronze, and total medals in the Summer Paralympic Games from 1992 to 2016. It was observed that several external factors could influence performance indicators, including an increase in the number of participants and greater and better government investment. Additionally, it was noted that preparation should be based on a multidisciplinary team, meaning that improvement in performance relies on knowledge and investment from governments [21].

Within this paralympic perspective, the participation of the Portugal team in the Tokyo 2020 Paralympic Games was evaluated through sociodemographic and psychosocial variables (positive and negative effects, life satisfaction, resilience, and social support). The Paralympic athletes presented high levels of life satisfaction, high positive affect, low negative affect, and substantial levels of resilience and social support, which appeared to be essential variables for these athletes [22].

A South Korean study also assessed the physical and sports needs of people with disabilities and how these needs should be reflected in policies and practices to improve their quality of life. The results indicated that para-sport projects should be community-focused, and the environment should be stable for the professionals involved, especially in sports facilities. Additionally, continuous capacity development should be prioritized. Facilities should be utilized more effectively by people with disabilities, and effective communication should be encouraged to help them understand the importance of physical activity and sports for their health [23].

In Brazil, the effect of different warm-up types on the strength and skin temperature of para-powerlifting athletes was evaluated. The results showed that there were no significant differences between warm-ups with stretching and specific exercises or the lack of engagement in warm-ups. However, thermal images demonstrated that traditional warm-up methods better suited the objectives of para-powerlifting [24]. The relationship between individuals with disabilities and the immune system was also assessed, including with respect to chronic low-grade inflammatory states and immunodepression. The impact of disability on various variables, ranging from physical fitness to well-being, quality of life, sleep, and nutritional aspects, among others, was examined. The study also analyzed the variability of parameters related to exercise/physical activity and the intra- and inter-individual variability of the immune response to exercise. Overall, moderate-intensity training was associated with optimal immunity and resistance to infections, such as upper respiratory tract infections, among athletes. Intense training periods with insufficient recovery times could lead to a temporary state of immunosuppression, which should resolve with a few days of rest/recovery from exercise [25].

Furthermore, research from Italy focused on para-rowing, wherein a scoping review enhanced by bibliometric analyses provided a comprehensive synthesis of knowledge related to the sport. The academic community of para-rowing was found to consist of 78 researchers, 16 (20.51%) of whom were highly interconnected. The study identified gaps in areas such as sports nutrition, doping, and psychological aspects among para-rowers who are not visually impaired [26]. The same Italian group evaluated paralympic powerlifting (PP) through a scoping literature review enhanced by a bibliometric analysis using large databases. The results indicated that the community studying the sport was poorly interconnected, with most authors contributing to only one article. However, one author was a central node in the author network, with 59.5% of the reviewed scientific material attributed to a Brazilian research group. The study suggested the need for increased connectivity within the research community and highlighted the significant growth potential of the sport [27].

In addition to these studies, other modalities were also investigated. For instance, badminton was evaluated through a visual reaction training system for improving footwork (VRTS) among badminton players. The results indicated that badminton footwork agility training through VRTS could enhance players’ skills and agility in this sport [28].

Environmental aspects were also studied, particularly training at different altitudes. Concentrations of erythropoietin (EPO), hemoglobin (Hb) levels, and VO2max values were assessed. Living and training at higher altitudes were associated with improvements in EPO, Hb, and VO2max compared to locations closer to sea level. Training at altitude was found to be favorable for enhancing sports performance [29].

In conventional swimming, the use of playful methods has been shown to have positive effects on sports learning. Two different swimming learning programs, one alternative and the other standardized, were evaluated. The alternative swimming learning program proved to be more efficient or equally effective compared to the standardized method regarding water skills, technique, swimming performance, and salivary cortisol concentration [30].

Basketball was also a subject of study, particularly the prevalence of back pain and musculoskeletal disorders and associated factors among basketball players. The corresponding study found a high prevalence of cervical pain, followed by lumbar and back pain, among basketball players. Preventive programs were recommended to improve the health and sports performance of these athletes [31].

Additionally, a study evaluated military personnel and their visceral adipose tissue (VAT) in relation to inflammatory processes. The study suggested that quantifying VAT could be used to estimate the risk of developing metabolic syndrome (MS). The results indicated that VAT ≥ 1025.0 cm^3^ (1086.0 g) was associated with MS risk factors and served as a predictor of the disease presenting good indicators of sensitivity and specificity [32].

In this context, this Special Issue covers current and varied topics, with broad relevance for both sports and occupational health, thereby making significant contributions to overall health. Furthermore, it was observed that there was a considerable body knowledge focused on people with disabilities, resulting in significant advancements in paralympic sports and the control of loads and variables related to this sector and thus contributing to the improvement of their life conditions and health.
